# Pancreatic metastasis from gastric carcinoma: a case report

**DOI:** 10.1186/1477-7819-2-43

**Published:** 2004-12-07

**Authors:** Moritz N Wente, Frank Bergmann, Boris E Fröhlich, Peter Schirmacher, Markus W Büchler, Helmut Friess

**Affiliations:** 1 Department of General Surgery, University of Heidelberg, Heidelberg, Germany; 2 Institute of Pathology, University of Heidelberg, Heidelberg, Germany

## Abstract

**Background:**

The pancreas is a rare but occasionally favored target for metastasis. Metastatic lesions in the pancreas have been described for various primary cancers, such as carcinomas of the lung, the breast, renal cell carcinoma and sarcomas.

**Case presentation:**

We report the case of a 60-year old female with a mass in the pancreatic head four years after partial gastrectomy for gastric adenocarcinoma. The patient underwent a pancreatoduodenectomy. Pathological examination revealed metastases of the primary gastric carcinoma within the pancreatic head and in regional lymph nodes.

**Conclusions:**

Pancreatic tumors in patients with a history of non-pancreatic malignancy should always be considered to be a putative metastatic lesion at an unusual site. If the pancreas can be identified as the only site of spread, radical resection may prolong survival.

## Background

The pancreas is an uncommon location for solitary metastasis from other primary cancers [1]. Despite this, in large autopsy series the prevalence of pancreatic metastasis has been described to be as high as 6% to 11% [2]. Whereas renal cell carcinoma appears to be the most common primary tumor to cause secondary pancreatic tumors, a variety of other cancers may spread to the pancreas, such as colon cancer, non-small cell lung cancer, and sarcomas [3]. This article presents the case of a pancreatic metastasis presenting as first site of gastric cancer recurrence four years after primary diagnosis.

## Case presentation

**Figure 1:**
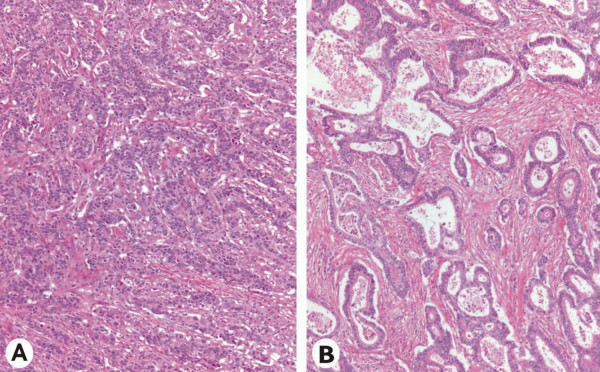
Histomorphologic appearance of the primary gastric carcinoma (A) and a paragastric lymph node metastasis (B). Photomicrograph shows that the primary tumor is mainly composed of solid and tubular formations, whereas, a marked desmoplastic stromal reaction is seen in the lymph node metastasis. (hematoxylin and eosin × 40).

A 60-year-old woman presented with elevated blood levels of the tumor markers CEA (17.3 μg/L, normal <2.5 μg/L) and CA 19-9 (121 U/ml, normal <37 U/ml). Four years before being referred to our institution, the patient had undergone gastric resection (Billroth II gastrectomy) for an adenocarcinoma of the stomach. The tumor was located at the lesser curvature of the gastric antrum, measuring 3 cm in the largest diameter. Pathologic examination revealed a gastric carcinoma of low differentiation, which infiltrated the gastric wall into the subserosal layer without penetrating the serosa. Microscopically, the carcinoma was mainly composed of tubular formations of mitotically active, atypical epithelial cells (Figure 1A). The tumor also displayed areas of marked desmoplastic stromal reaction, as well as areas of rather glandular differentiation. The latter two were mainly observed in paragastric lymph node metastases (Figure 1B). The carcinoma at stage pT2 pN1 (6/15) M0 G2 was completely resected (R0 resection). No recurrence was detected during the regular follow-ups.

**Figure 2:**
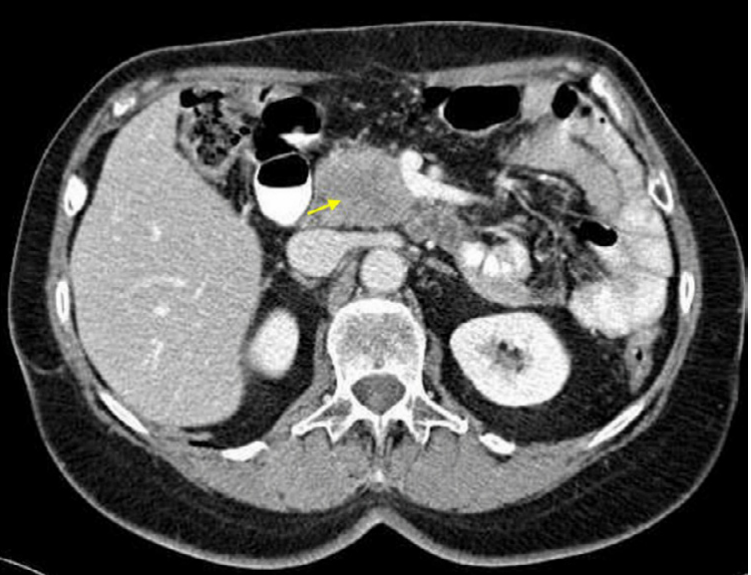
Computed tomography of the abdomen four years after Billroth II resection for gastric cancer, revealing an inhomogenous mass in the pancreatic head, 4 cm in diameter. (Picture courtesy the Division of Radiology, German Cancer Research Center, provided by PD Dr. med. S. Delorme).

**Figure 3:**
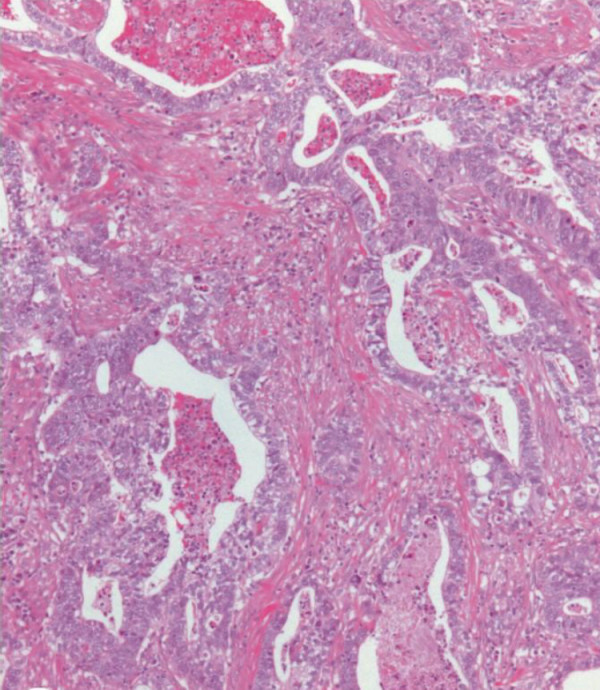
Histomorphologic appearance of the delayed pancreatic metastasis (hematoxylin and eosin × 100). As in the lymph node metastasis, a marked desmoplastic stromal reaction is seen in the pancreatic metastasis.

Four years after gastric resection, however, ultrasound examination, computed tomography and magnetic resonance imaging revealed an inhomogeneous mass of the pancreatic head, measuring 4 cm in largest diameter (Figure 2). Radiographically, no other masses were detected. For differential diagnosis, a primary carcinoma of the pancreas and a metastasis of the gastric carcinoma were considered. Following explorative laparotomy, the pancreatic mass was resected performing a partial pancreatoduodenectomy with resection of the distal bile duct (Whipple's procedure). Furthermore, the former gastroenterostomy was resected and revised. On pathologic examination, the tumor of the pancreatic head grossly presented as white to yellowish, firm mass. Microscopically, the tumor consisted of solid and glandular formations of atypical epithelial cells with distinct nuclear pleomorphism and presented marked desmoplastic stromal reaction, as well as areas of necrosis (Figure 3). The duodenal wall and the peripancreatic tissue were infiltrated by the tumor. Lymph node metastases were detected in two peripancreatic lymph nodes. The histomorphological appearance of the pancreatic tumor was in good accordance to some areas of the primary gastric tumor, and especially the growth pattern found in the paragastric lymph node metastases coped well with the microscopic picture found in the pancreatic tumor. Immunohistochemical analyses revealed identical expression patterns in the gastric carcinoma and the pancreatic mass, both displaying positive reactions with antibodies towards cytokeratins 8, 18 and 19, as well as carcinoembryonic antigen (CEA), whereas no reactions were seen with antibodies towards cytokeratins 7 and 20. Because of these findings and due to the lack of pancreatic cancer progenitor lesions, pancreatic intraepithelial neoplasias (PanINs), within the non-neoplastic pancreatic tissue of the Whipple's resection specimen, the pancreatic tumor and the two regional lymph node metastases were considered to be metastases of the primary gastric carcinoma.

The patient was discharged from the hospital without any perioperative morbidity on the ninth postoperative day. The postoperative blood levels of the tumor markers declined to normal values (CEA 2.6 μg/l, CA 19-9 24 U/ml). Due to the complete surgical resection and the lack of risk factors for recurrence, the patient received no further adjuvant therapy. Under regular follow-up for one year with determination of the tumor markers and computed tomography, the patient revealed no signs or symptoms of local or systemic recurrence.

## Discussion

Death from recurrence of gastric adenocarcinoma occurs in 70–75% of patients during the first two years after surgical intervention, however, reports of recurrences more than 10 years after primary diagnosis have been reported as well [4]. The most frequent sites of tumor recurrences include local, regional and peripheral lymph nodes, as well as the liver, the lungs, and the peritoneum [5]. Furthermore, solitary metastasis in other organs, such as the thyroid gland or the spleen have been described [6,7]. In contrast to direct infiltration into the pancreas, metastases of gastric cancer into the pancreas are considered to be extremely rare and to our knowledge only four cases have been reported in the English literature [8-10].

Adenocarcinomas of the pancreas and of other primary sites frequently display a large histomorphological and immunohistochemical overlap. Thus the differential diagnosis of primary pancreatic cancer versus solitary metastases of other adenocarcinomas may be very difficult – if not impossible – using common pathological and immunohistochemical techniques. According to Robbins *et al *[3], solitary pancreatic masses can be classified as secondary tumors to the pancreas in only 2% of the cases, and they are frequently misdiagnosed as primary pancreatic cancers. As a consequence from this, the subtle diagnostic work-up for isolated masses in the pancreas needs to inherit a meticulous elaboration of the medical history of the patients, in particular focused on previous non-pancreatic malignancy.

Pancreatic resections can nowadays be performed with low morbidity and mortality rates, in particular in high-volume centers [11,12]. Results of surgical extirpation of isolated metastases to the pancreas from various primary tumors provide improvement with regard to long-term survival [1,2]. Therefore, a resection of isolated metastases in the pancreas should be considered as a treatment option in patients with the history of non-pancreatic malignancy [13].

## Competing Interests

The authors declare that they have no competing interests.

## Authors' Contributions

**MNW **collated the information, searched the literature and wrote the manuscript.

**FB **and **PS **contributed to the pathological aspects of the manuscript and helped in preparing the manuscript.

**BEF **assisted in literature search and writing of the manuscript.

**MWB **and **HF **managed the patient and helped in preparing the manuscript and edited the final version with **PS**.

All authors read and approved the final version of the manuscript.
